# Research on Remaining Useful Life Prediction of Equipment Based on Digital Twins

**DOI:** 10.3390/s26041240

**Published:** 2026-02-13

**Authors:** Jiaju Wu, Yuanlin Zhou, Xiaodong Wang, Chuan Chen, Yongqi Ma, Chunrui Zhang

**Affiliations:** 1 Institute of Computer Application, China Academy of Engineering Physics, Mianyang 621900, China; wujj@caep.cn (J.W.);; 2 College of Civil Aviation, Nanjing University of Aeronautics and Astronautics, Nanjing 210016, China

**Keywords:** remaining useful life prediction, digital twin, integrated learning, genetic algorithm, engineering implementation

## Abstract

Remaining Useful Life (RUL) prediction is a key factor in fault diagnosis, prediction, and health management (PHM) during equipment operation and service. Its purpose is to predict the time interval from the current moment to the complete failure of the equipment, serving as the basis for condition-based maintenance strategies. Effective RUL prediction enables the scheduling of maintenance plans in advance, thereby reducing equipment downtime and safety incidents. The RUL prediction of equipment and its critical components is an important means of fault diagnosis and prediction. Real-time and accurate RUL prediction results are prerequisites for implementing preventive maintenance, condition-based maintenance, and failure-based maintenance strategies, allowing the identification of optimal maintenance timing. This constitutes a crucial aspect of precise equipment support. The real-time, high-efficiency communication of digital twin technology can support real-time online RUL prediction for equipment. This paper introduces digital twin technology and constructs a digital twin-based RUL prediction model for equipment. The study proposes an integrated learning-based RUL prediction method for equipment, validated through experiments to demonstrate its accuracy and robustness. Finally, this paper presents an engineering implementation plan for online RUL prediction of equipment based on digital twins.

## 1. Introduction

There are many model-based methods for RUL prediction of equipment systems and components, and some studies use equipment monitoring data to predict their remaining useful life. With the gradual improvement of continuous detection and monitoring capabilities throughout the entire lifecycle of equipment, a large amount of monitoring and detection data is driving the widespread application of data-driven RUL prediction methods in the field of equipment. Data-driven prediction methods, especially deep learning (DL) models, have rapidly developed due to their versatility. They extract degradation features from real-time twin data of equipment nonlinearly, construct mathematical models that approximate degradation laws, establish a mapping relationship between sensor values and system health status, and achieve end-to-end RUL prediction of the time or period when equipment reaches the failure threshold from the current moment. This has gradually become the mainstream research in this field. At present, data-driven equipment RUL prediction mostly adopts single time series prediction models, such as vector regression, Random Forest, multi-layer perception, etc. It is difficult for a single machine learning algorithm to accurately predict the Remaining Useful Life of equipment. In addition, in the competition among numerous machine learning algorithms, it is difficult to choose a single algorithm or model to accurately predict the RUL of equipment. Moreover, the portability of a single method is often only able to adapt to a single dataset, and the robustness of prediction methods is low.

The development and application of digital twin technology make it possible to accurately map physical equipment with digital twins. Equipment users are no longer satisfied with mapping physical equipment structures to geometric models and behavioral models to equipment movements. They hope to use twin data mining to uncover potential patterns and more accurately reflect the evolution process of the health status of physical equipment, enabling precise mapping of equipment physical entities [1,2,3]. The real-time and efficient communication of digital twins can support RUL online prediction. The RUL prediction driven by twin data is essentially data-driven, but the scope and quantity of twin data are larger than traditional monitoring data, and the accuracy of prediction results is high. The prediction results need to be fed back in real-time to physical equipment and virtual digital equipment. The data scope of twin data can be expanded from traditional physical equipment monitoring/testing data to historical data, quality data, geometric data, performance data, monitoring/testing data, virtual digital equipment data, and virtual real fusion data of physical equipment.

This paper introduces digital twin technology and proposes a prediction model for the Remaining Useful Life of equipment based on digital twins. This paper designs a residual service life prediction process based on a twin data-driven approach. On this basis, a twin data-based integrated learning RUL prediction method is proposed to address the low accuracy and robustness of a single algorithm. The method simulates the degradation process of key equipment components and predicts their Remaining Useful Life. Finally, this article presents the implementation process of online engineering for predicting RUL based on digital twins. The integrated learning method integrates the results of multiple basic learners for predicting the Remaining Useful Life of equipment, reducing the proportion of poorly performing methods and increasing the proportion of better performing methods, resulting in the optimal implementation of the overall model. The integrated learning method consists of five basic learners: Relevance Vector Machine (RVM), Random Forest (RF), Elastic Net (EN), Autoregressive (AR) model, and Long Short-Term Memory (LSTM) neural network. The Genetic Algorithm (GA) is applied to integrated learning methods, finding and determining the optimal weights of the base learners, and obtaining the final prediction results of equipment systems or components [4].

## 2. Related Works

Remaining Useful Life (RUL) prediction is one of the core elements of equipment condition-based maintenance decision-making, providing a decision-making basis for predictive maintenance, ensuring equipment availability, improving utilization, and reducing maintenance costs [5]. RUL is affected by operating conditions and environment, and varies with the time of equipment operation and use. It is defined as shown in Equation (1):

(1) $$RUL\left(t\right)=\left\{T_{f}\left(t\right)-t|T_{f}\left(t\right)\geq t,M\left(t\right)\right\}$$

In the equation, *t* is the current time, $T_{f}\left(t\right)$ is the fault time, and $M\left(t\right)$ is the operating condition at time t.

RUL prediction includes three mainstream methods: physical modeling, data-driven, and statistical analysis. RUL prediction based on physical modeling uses physical models to characterize the relationship between detection/monitoring data and RUL. The prediction results are interpretable, do not require a large amount of data, and have high generalization ability, accuracy, and credibility. However, there are serious issues, such as relying heavily on physical or domain knowledge. Common models for predicting RUL based on physical models include the Paris model for fatigue crack growth, calendar aging, and cyclic aging models for batteries, and dynamic models for gears. Shen et al. [6] constructed a family of health evaluation indicators based on generalized power mean and high-order origin matrix, and proposed an improved Paris Erdogan model that takes the optimal health indicator as input to predict the RUL of bearings after initial failure. Li et al. [7] analyzed the advantages and disadvantages of physical models and data-driven Remaining Useful Life prediction, proposed a RUL prediction that integrates physical models and data-driven models, and reviewed the research progress of data-driven RUL prediction based on physical models.

Data-driven RUL prediction generally relies on machine learning as its core, which discovers the degradation trend of equipment and components through data processing and analysis, identifies performance change patterns, and does not require a deep understanding of physical mechanisms. It has heuristic characteristics and has certain limitations for small sample degraded data [8]. At the same time, data-driven models and data are related to the calculation of large amounts of data and complex models, which require high hardware performance. Vaishali et al. summarized the application of machine learning in predicting the capacitance and Remaining Useful Life of supercapacitors, including convolutional neural networks (CNNs), recurrent neural networks (RNNs), linear regression, decision trees, vectorizers, transfer learning, Random Forests, and other methods [9]. Zheng Jianfei et al. [10] proposed a multi-level degradation bearing remaining service life prediction method based on a TBiGAN parallel network. Zhou Yixin et al. [11] proposed a transformer-based hybrid method with multiple features for lithium battery Remaining Useful Life prediction.

Statistical analysis is a traditional method for life prediction, applied to diagnosis and prediction in multiple fields such as machinery, electronics, and complex systems, including Weibull analysis, particle filtering, etc. Lu et al. proposed an accelerated failure time regression residual service life prediction model based on Weibull, which improved the prediction efficiency for large amounts of data [12]. Yu et al. proposed a method for predicting the RUL of hydraulic pumps, using multivariate fusion and monotonic constrained particle filtering [13].

The development of digital twin technology has brought new research ideas for equipment RUL prediction. Twin data comes from the physical space of equipment, virtual digital space, and the process of virtual real interaction fusion, including equipment physical mechanism models, behavior models, rule constraint models, real-time monitoring data, simulation data, optimization control data, historical monitoring data, experimental data, etc. Twin data has a wider range and larger quantity compared to traditional equipment condition monitoring data. Data-driven RUL prediction, which extends from traditional state monitoring data to twin data, can predict equipment RUL from multiple dimensions. Weighted calculation of the impact weight of each dimension on the remaining life can more accurately predict the RUL value of equipment. Meng proposed a data/physics-driven digital twin model to predict the Remaining Useful Life of rolling bearings [14]. He used theoretical damage mechanics to describe the fatigue damage evolution process of rolling bearings throughout their entire life cycle and established a delamination fatigue evolution model. He introduced the fatigue defects predicted by the delamination fatigue evolution model into the dynamic model and constructed a high-fidelity model for bearings. Based on this, a local-to-global dynamic update framework was designed for the interactive coupling of real-time monitoring data and corresponding simulation data of rolling bearings, as well as the updating of high-fidelity model parameters. Finally, neural networks were used to predict the RUL of rolling bearings.

To supplement the diagnosis of actual equipment system faults and support maintenance decisions with RUL prediction results, the research questions for RUL prediction are as follows. (1) The extraction of temporal features that meet the practical needs of industrial applications is required. Most equipment operates under complex working conditions such as high load, high temperature, high humidity, high salt, and dust. The data features have nonlinear, non-smooth, non-Gaussian, and low signal-to-noise ratio characteristics, requiring the extraction of high-quality temporal features with trend and monotonicity. The method needs to meet real-world environments and have high robustness. (2) Interpretable prediction: Although advanced achievements have been made in the field of RUL prediction based on machine learning, the “black box” characteristics limit its applicability in industrial scenarios. (3) The transferability evaluation from the source domain to the target domain: Most RUL prediction methods directly learn and transfer knowledge from the source domain, and need to be evaluated to determine whether the knowledge transformed from the “source domain -> target domain” is suitable. (4) The stability and reliability of prediction models are mainly focused on predicting accurate residual time values, and the uncertainty limits and confidence of prediction results need to be evaluated before they can be widely used in the industry. (5) Data privacy protection is extremely important in the field of equipment, and it is necessary to introduce federated learning and blockchain technology to address privacy protection issues. (6) RUL prediction under multiple failure modes is required, as internal random factors and complex external environments can lead to different failure modes and degradation processes in actual equipment system applications. Therefore, when predicting RUL, it is necessary to comprehensively consider the impact of various degradation factors on the lifespan. (7) Real-time online RUL prediction [15,16]. The interpretability, realistic display, high confidence, and high real-time performance of digital twins provide new solutions for the related problems of remaining life prediction. Combined with machine learning and digital twin technology, it can promote the application of Remaining Useful Life prediction in industrial scenarios.

## 3. Equipment Remaining Life Prediction Based on Digital Twins

### 3.1. Equipment RUL Prediction Model and Process Based on Digital Twin

**Figure 1:**
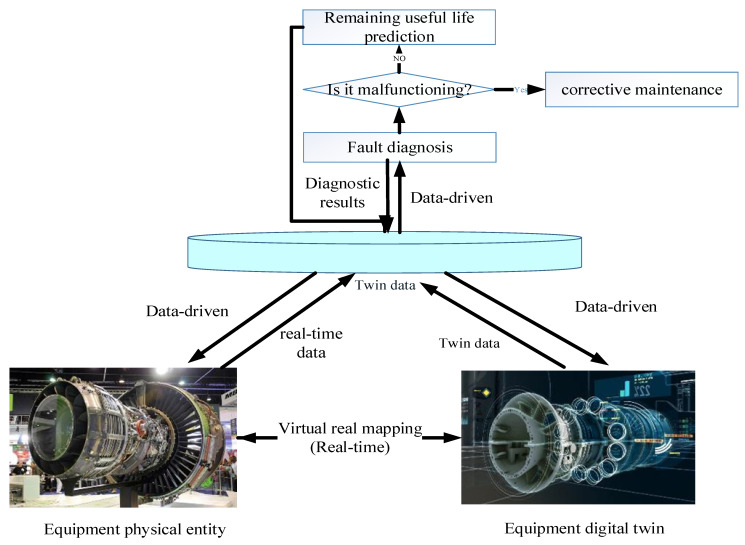
Equipment RUL prediction model based on digital twins.

The Remaining Useful Life prediction model for equipment based on digital twins is shown in Figure 1. This model is generally developed on the basis of fault diagnosis, mainly predicting the Remaining Useful Life of equipment that has begun to degrade but has not yet malfunctioned, achieving real-time online calculation of RUL, and outputting results as the basis for maintenance decisions. Based on the Remaining Useful Life prediction service, for equipment and its components that can operate normally but begin to degrade, the Remaining Useful Life of the equipment is predicted through real-time twin data, simulation data, etc. The Remaining Useful Life prediction results are also fed back to update the virtual digital twin of the equipment.

The RUL prediction service is online, real-time software based on twin data, such as equipment historical data, real-time information, model data, and simulation data, as well as various RUL prediction algorithms. The software is provided as a service, taking real-time twin data of the equipment as input, and uses a certain algorithm to calculate and predict the time from the current moment to the failure of the equipment, which provides feedback on the RUL to the digital twin of the digital space. The virtual digital twin displays the degradation process and Remaining Useful Life data of the equipment in a highly realistic manner, updates the virtual digital twin, and presents the degradation work of the equipment in real-time and high-fidelity. Equipment virtual digital twins determine whether preventive maintenance measures should be taken based on the set RUL threshold. When the RUL of the equipment is less than or equal to the set threshold, preventive maintenance measures are taken, and maintenance decision data is fed back to the equipment users in the physical space.

**Figure 2:**
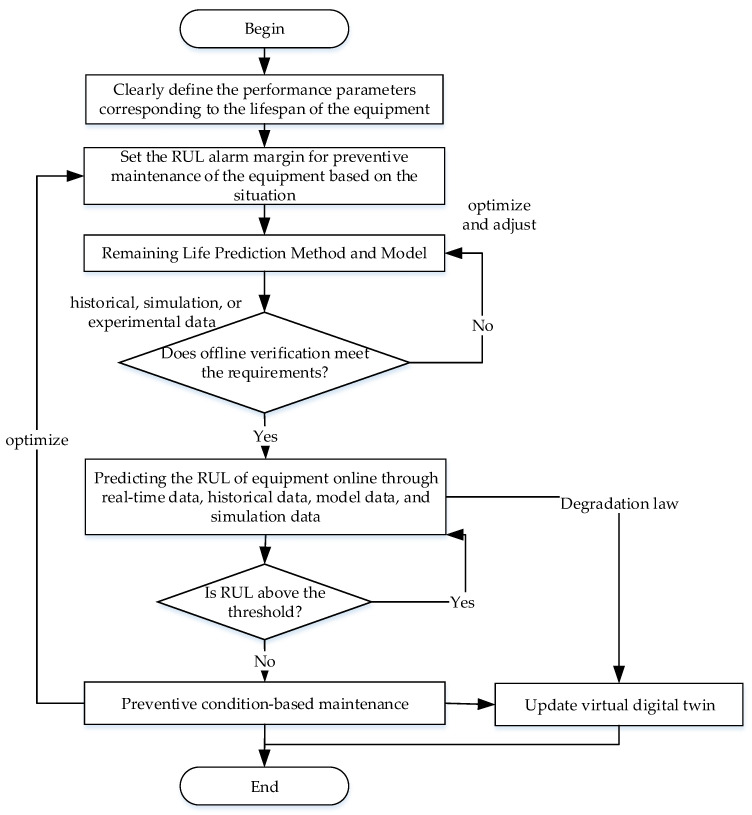
Equipment RUL prediction process based on twin data.

The process of predicting the RUL of twin data-driven equipment is shown in Figure 2, which is mainly used for equipment and its components that have degraded but are still operating normally.

Firstly, based on the degree of impact of parameters on the operational functions and performance of equipment, determine the main performance parameters that affect the lifespan of the equipment. Next, set the RUL threshold for condition-based maintenance. Then, choose prediction methods and models, such as statistical analysis, data-driven, physical models, or a combination of both. Furthermore, evaluate offline whether the prediction method meets the requirements through historical data, simulation data, or experimental data. If the requirements are not met, optimize and adjust the prediction method. If the requirements are met, real-time data, historical data, simulation data, and model data will be integrated, and the Remaining Useful Life prediction method will be used to calculate the RUL of the equipment and its components in real time. Finally, based on the set preventive maintenance life threshold, it is determined whether to continue monitoring the operation of the equipment or adopt a preventive maintenance strategy. At the same time, the RUL data, degradation patterns, and adopted strategies will be fed back to the virtual digital twin of the equipment, updating the virtual digital twin and displaying the degradation process and RUL of the equipment with high realism.

The twin data required for predicting the RUL includes equipment static attribute data, equipment real-time status data, environmental and operational data, maintenance and historical data, etc. Equipment static attribute data is used to construct an initial twin model and establish a predictive baseline, including equipment unique identification, model, design parameters, material attributes, and historical configuration change records. The real-time status data of equipment includes working condition data reflecting the real-time health status of equipment, which is the source of degradation feature extraction, including speed, load, pressure, temperature, voltage/current, vibration (time domain, frequency domain), acoustic emission, oil analysis (abrasive particles, viscosity), thermal imaging, and other status monitoring data. Environmental and operational data are used to evaluate external stress, identify operating conditions and load spectrum analysis, correct the impact of the environment on degradation models, including environmental temperature, humidity, dust, geographic location, equipment start stop sequence, working mode, operating instructions, etc. Maintenance and historical data are used for model training, validation, associating states with final life, and achieving iterative optimization of the model, including historical fault records, repair work orders, replacement parts lists, manual inspection reports, group operation data of similar equipment, and other historical data.

Data flows from physical equipment to digital twin platforms, usually through the path of “sensors/PLCs -> edge gateways -> cloud/ servers”, with different protocols used at different levels. From sensors, PLC, common protocols for reading raw data from underlying devices, such as CNC systems, include OPC UA, Modbus (RTU/TCP), CAN Bus, and dedicated controller protocols. The OPC UA protocol is the absolute mainstream and future direction of industrial interconnection. The Modbus (RTU/TCP) protocol is a simple and widely used serial communication protocol, commonly used in PLCs and instruments. CAN Bus is a standard protocol used in the internal networks of mobile devices such as automobiles and aviation, enabling communication between ECUs (Electronic Control Units). The dedicated controller protocol is used to communicate with specific brands of controllers, such as Siemens’ S7 protocol, Mitsubishi’s MC protocol, etc.

### 3.2. Integrated Learning Method for Equipment Remaining Life Prediction Based on Genetic Algorithm

To address the robustness and accuracy issues of a single method in online real-time RUL prediction, a residual service life prediction scheme based on integrated learning was designed. The integrated learning prediction method combines various basic learners and achieves optimal results through weighting. Due to the fact that different basic learners can obtain different results, the results can be merged to compensate for the shortcomings of a single basic learner. Usually, integrated learning has high learning ability and performance. Generally speaking, the greater the difference between the basic learners of integrated learning, the stronger the generalization of the model. That is to say, basic learners have less relevance, and the advantages of integrated learning will be more prominent. So, in order to improve the prediction accuracy of integrated learning algorithms, the basic learner algorithms need to be diversified.

The prediction methods based on integrated learning include two methods: sequential integration and parallel integration.

Sequential integration’s trainer uses the dependency relationship between basic trainers as the generation order principle, and generates the basic trainers participating in the training in order. The comprehensive trainer trains them one by one in order, so their performance can be understood in order, and their weights can be determined. Through iteration, the results become increasingly accurate. Inspired by feedback ideas, higher loss weights can be set for the initial base training of incorrectly predicted data. By repeating this operation, the optimal solution for the weight of each trainer in the sequence can be obtained. However, the results of the sequential ensemble method mainly rely on the performance of the first basic learner. Since it cannot be proven that the selected first basic learner performs the best, different training trainers can be compared as the first basic learner to determine the best one.

In parallel integration, to utilize the independence of the base learners, the participating base learners are merged into the parallel learners. Unlike sequential integration, integration can be achieved by independently adjusting the weights of the base learners. The dependency between basic learners is very low, so there is no need to consider correlation. Even if the prediction result of one learner is poor, other better learners can reduce its adverse effects. Parallel integration can reduce prediction errors and significantly improve the accuracy of results.

**Figure 3:**
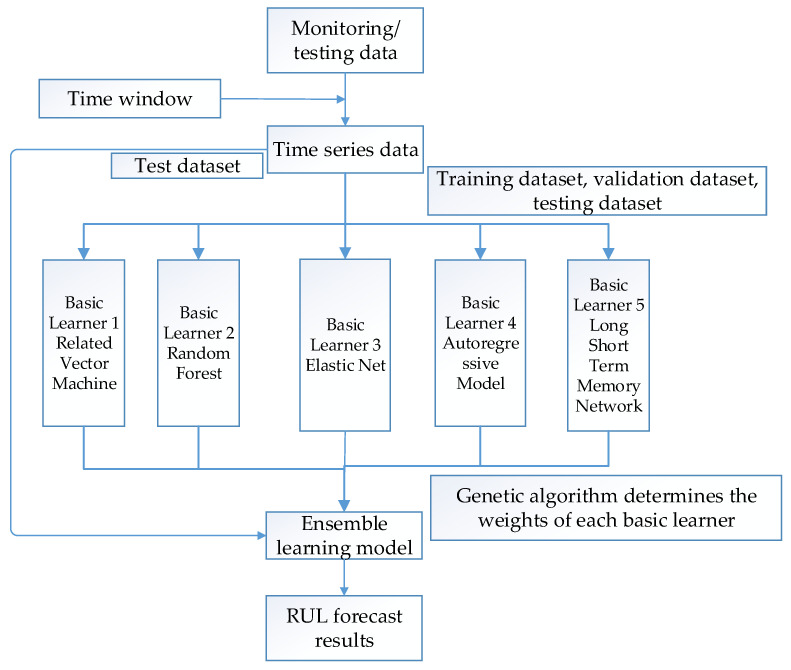
Twin data-driven RUL integrated learning method.

The overall performance of sequential ensemble methods overly relies on the stability of the first base learner, while the parallel integration methods are more independent of the base learner and can solve the problem of improper selection of the first base learner. Therefore, the parallel integrated learning method is adopted as shown in Figure 3.

The Integrated learning model consists of three parts: raw data time series conversion, basic learner, and ensemble model. Firstly, extract a time series from the raw data that can characterize the degradation index, and convert it into supervised learning data according to a fixed time window. Then divide the experimental data into three subsets: training, validation, and testing. The training dataset is fed into each base learner for supervised learning, while the validation dataset is used for better parameters. After training and validation on the dataset, the trained model forms the training model. Next, put the test dataset into the training model and calculate the RUL value. Finally, a genetic algorithm is used to minimize the LOSS-MSE between the predicted and actual results, in order to determine the optimal weights for each base learner. The calculation formula for LOSS-MSE is shown in Equation (2).

(2) $${LOSS}_{\_MSE}={\frac{1}{n}\sum_{i=1}^{n}\;(y_{i}-ay_{i}^{1}-by_{i}^{2}-cy_{i}^{3}-dy_{i}^{4}-ey_{i}^{5})}^{2}$$

In Equation (2), *n* represents the number of data sizes. The $y_{i}^{j}$ represents the prediction result of sequence i for base learner j (j = 1, 2, 3, 4, 5). The $y_{i}\;$ represents the actual value of sequence i. And *a*, *b*, *c*, *d*, and *e* represent the weights of each base learner, respectively. Finally, based on the optimal weights obtained by the genetic algorithm for each basic learner, the RUL prediction value of the equipment is calculated, as shown in Equation (3).

(3) $${\;y}_{i}=ay_{i}^{1}+by_{i}^{2}+cy_{i}^{3}+dy_{i}^{4}+ey_{i}^{5}\;(i=1,2,\ldots ,n)$$

The basic learner of the Integrated learning algorithm proposed in this article includes five different types of time series prediction algorithms: Related Vector Machine (RVM) [17], Random Forest (RF) [18], Elastic Net (EN), Autoregressive (AR) model, and Long Short-Term Memory (LSTM) network [19].

The theories and mechanisms of the five basic learners mentioned above are as follows:

1. *Bayesian*-based RVM model

The Bayesian-based RVM model is a sparse probability model. Its core advantages are highly compatible with the demand predicted by RUL. It is able to quantify uncertainty. Unlike traditional models that require additional calculation of confidence intervals, RVM directly provides a complete predicted probability distribution. This is crucial for evaluating the reliability of RUL predictions and developing risk contingency plans. RVM has excellent generalization and sparsity. Through the “automatic correlation determination” mechanism, RVM can automatically screen the most critical features for prediction (such as specific vibration frequencies), effectively preventing overfitting. It is very valuable in industrial scenarios with scarce fault samples and high data noise. RVM implements probabilistic output, which is a posterior distribution that conforms to Bayesian rules, making it easier to integrate more complex Bayesian dynamic models (such as filtering algorithms used for state estimation) for continuous updates. In situations where equipment data is limited (such as new or expensive equipment), feature dimensions are high (such as multi-sensor fusion) and decision-making heavily relies on risk probabilities (such as predicting key component failures); the probability output provided by Bayesian-based RVM models can seamlessly integrate with digital twins for visual risk warning.

RVM is a Bayesian sparse computing method used for regression and classification. It is consistent with support vector machine functions, has stronger generalization ability, does not require cross-validation of hyperparameters, and the kernel function can be arbitrarily specified and not positive definite. The solution is sparser than SVM [20,21]. The prediction result of RVM is obtained from Formula (4).

(4) $$y\left(x\right)=w^{T}\Phi \left(x\right)+b$$

In the equation, Φ (x) is the kernel function, and x is the input. $w^{T}$ represents the parameter matrix. b represents deviation. The conditional probability distribution of t is shown in Formula (5).

(5) $$p\left(t|x,w,\beta \right)=N\left(t|y\left(x\right),\beta^{-1}\right)$$

Among them, β = σ − 2 is the noise accuracy (reciprocal of noise variance), σ is the accuracy of parameter w, and N() is a Gaussian distribution.

RVM is a Bayesian method that maximizes the edge likelihood function to solve hyperparameters, defined in Formula (6).

(6) $$p\left(t|x,\;\alpha ,\;\beta \;\right)=\int p\left(t|x,\;w,\beta \;\right)p(w|x)dw$$

2. Random Forest Model Based on Ensemble Tree

Random Forest, as a comprehensive training method, includes various regression decision trees. Each regression decision tree in the forest is independent. The final value of RF is determined by the values of each regression decision tree, and the output is the average of the outputs of each regression decision tree [22]. The randomness of RF is reflected in two aspects:

(1) The sample is random. Each regression decision tree root node sample is randomly obtained from the training set.
(2) The features are random. Randomly select candidate features and use the most suitable features as segmentation nodes to construct each regression decision tree.

Randomness is used to represent the uncertainty of training and prevent overfitting of the method. In the process of training and optimizing the regression decision tree, it is equivalent to optimizing the following Formulas (7) and (8).

(7) $${\;argmin}_{x,\;v}G\left(x,\;v\right)$$

(8) $$G\left(x,\;v\right)=\frac{1}{N}\left({\sum_{y_{i\in X_{left}}}\left(y_{i}-y_{left}\right)}^{2}+{\sum_{y_{j\in X_{right}}}\left(y_{j}-y_{right}\right)}^{2}\right)$$

3. EN model based on regression analysis

A linear model that uses both L1 and L2 penalty terms in the objective function of the Elastic Net (EN) based on regression models. L1 refers to the Least Absolute Shrinkage and Selection Operator (LASSO), while L2 refers to Ridge regression. When there are many variables in the data, and you want to extract some feature variables from them, LASSO can meet your needs. Ridge regression may be more suitable when each variable in the data is particularly important, and one does not want to eliminate certain variables. Therefore, EN combines the advantages of LASSO regression and ridge regression by adding L1 and L2 regularization penalty terms in the process of minimizing the objective function. The objective function is shown in Equation (9).

(9) $${\;min}_{w}\sum_{i=1}^{N}{\left(y_{i}-{w^{T}x}_{i}\right)}^{2}+\gamma \rho {\left\|w\right\|}_{1}+\frac{\gamma (1-\rho )}{2}{\left\|w\right\|}_{2}^{2}$$

4. AR model based on a random model

AR is a statistical-based Autoregressive model commonly used to solve time series prediction problems. AR is developed from linear regression, and its mathematical expression is shown in Formula (10).

(10) $$y\left(t\right)=\sum_{i=1}^{n}a_{i}y\left(t-i\right)+e\left(t\right)$$

Here, n represents n-order autoregression.

The goal of model derivation is to increase the effective data. In AR models, the independent variable is not used to predict the dependent variable, but to predict the independent variable itself. The prerequisite for Useful AR is variable autocorrelation. For example, the remaining capacity of a battery is self-correlated with charging and discharging cycles, while the lifespan of an engine is self-correlated with flight cycles or flight time. The commonly used Yule–Walker equation form is used to solve the AR order [23].

5. LSTM model

**Figure 4:**
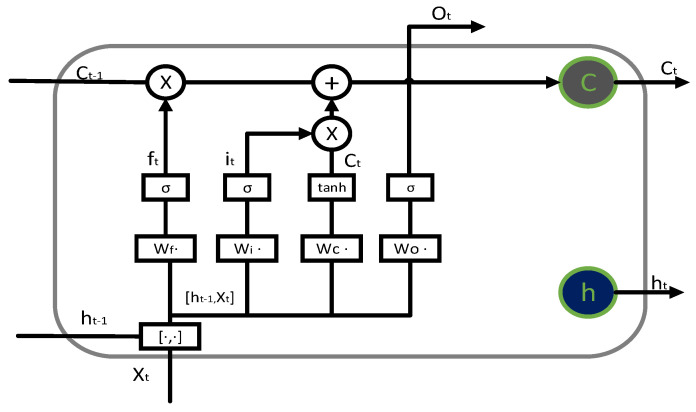
LSTM structure diagram.

A recurrent neural network (RNN) is an artificial neural network (ANN) that processes time series data. However, the difficulty in handling long-term dependencies and the explosion or disappearance of gradients are the challenges in the application of RNNs. LSTM, as a special RNN, adds cell states to effectively handle the long-term dependency problem of RNNs. It also adds a forget gate to effectively solve the gradient vanishing phenomenon. Forget and input–output gates are useful to control cell states [24,25]. The internal structure of LSTM, as shown in Figure 4, adds unit state $C_{t}$ to the RNN, making it store time series data for a longer period of time. By stacking LSTM units, a multi-layer LSTM network is constructed to mine time series information in the data and predict the results. The relevant calculation formulas are shown in Formulas (11)–(18).

The forget gate uses a sigmoid unit to process the pre-time $h_{t-1}$ and the current input $h_{t-1}$, determining the cell state $C_{t-1}$ to retain information, as shown in Equations (11) and (12):

(11) $${\;f}_{t}=\sigma \left(W_{f}\cdot \left[h_{t-1},\;x_{t}\right]+b_{f}\right)$$

(12) $$\sigma \left(x\right)=\frac{1}{1+e^{-x}\;}$$

In the formula, $b_{f}$ is the forget gate bias term, and Formula (11) is the sigmoid calculation.

The input gate adopts a sigmoid unit to determine how much information to update, where the updated information is obtained by processing the information of $h_{t-1}$ and $x_{t}$ by the tanh unit, as shown in Equations (13)–(15).

(13) $${\;i}_{t}=\sigma \left(W_{i}\cdot \left[h_{t-1},\;x_{t}\right]+b_{i}\right)\;$$

(14) $${\tilde{C}}_{t}=\tanh\left(W_{C}\cdot \left[h_{t-1},\;x_{t}\right]+b_{C}\right)$$

(15) $$\;\tanh\left(x\right)=\frac{e^{x}-e^{-x}}{e^{x}+e^{-x}}\;$$

Among them, $b_{i}$ and $b_{c}$ represent the bias term, and Equation (15) denotes the computation operation of tanh. Then, $C_{t-1}$ is updated from the input gate and forget gate to $C_{t}$. Formula (16) represents the update from $C_{t-1}$ to $C_{t}$.

(16) $${\;C}_{t}=f_{t}*\;C_{t-1}+i_{t}*{\tilde{C}}_{t}$$

Finally, the final $h_{t}$ is determined based on $C_{t}$ output gates $h_{t-1}$ and $x_{t}$, as shown in Equations (17) and (18).

(17) $$o_{t}=\sigma \left(W_{o}\cdot \left[h_{t-1},\;x_{t}\right]+b_{o}\right)$$

(18) $${\;h}_{t}=o_{t}*\tanh\left(C_{t}\right)$$

In the formula, * represents the Hadamard product, and $h_{t}$ enters the next layer.

### 3.3. Data Preprocessing

(1) Feature parameter extraction

The first step in predicting the RUL of equipment is to extract the fault characteristic parameters from the real-time twin data of the equipment. Fault characteristic parameters are divided into three categories: time domain, frequency domain, and time–frequency domain. The purpose of feature parameter extraction is to obtain a feature dataset that is sensitive to the impact on the RUL of equipment. The feature parameters are sorted by the weighted sum of the three indicators of time correlation, monotonicity, and robustness, and the parameters with stronger influence are selected as the RUL prediction parameter set. The calculation formula is shown in Equation (19).

(19) $$C_{pi}=\omega_{1}*Rel\left(P,t\right)+\omega_{2}*Mon\left(P\right)+\omega_{3}*Rob(P)$$

In the formula, $C_{pi}$ is the characteristic index of parameter P; $\omega_{1}$, $\omega_{2}$, and $\omega_{3}$ are the evaluation weights of each evaluation parameter, which can be set to 0.3, 0.5, and 0.2; Rel (P, t) is the correlation of the parameter over time; Mon (P) is the monotonicity of the parameter value; and Rob (P) is the robustness of the parameter data.

(2) Time series conversion

To achieve supervised learning for each basic learner, this paper converts the degradation index extracted from the raw data into a supervised learning sequence with a fixed time window. Time series conversion refers to the use of methods such as data smoothing, differential calculation, decomposition, aggregation, and discretization, stationarity conversion, alignment, or padding to transform initial data into time series data, facilitating feature acquisition. This article conducts supervised training based on time series representing degradation indices and abstracts the RUL prediction of the equipment as a time series prediction of reduced order index. Among them, the time window is defined as the time when the next cycle value was predicted in the past, as shown in Equation (20).

(20) $${\;RUL}_{t}=f\left({RUL}_{t-1},\;{RUL}_{t-2},\;\ldots {RUL}_{t-L}\right)$$

In the formula, f represents the base learner, ${RUL}_{t}$ represents the RUL value at time t, and L represents the size of the time window.

### 3.4. Model Evaluation Indicators

Relative error (RE), mean absolute error (MAE), and root mean square error (RMSE) are generally used to evaluate the robustness and performance of RUL prediction algorithms [26,27]. Therefore, RE, MAE, and RMSE are selected as evaluation metrics for RUL prediction methods to assess the performance and robustness of individual base learners and the integrated learning method described in this paper. Generally, if the RE, MAE, and RMSE values are small, the RUL prediction algorithm has better robustness and accuracy. The calculations of RE, MAE, and RMSE are shown in Equations (21)–(23).

The calculation formula for RE is as follows:

(21) $$RE=\frac{\left|\hat{y}-y\right|}{\left|y\right|}$$

The calculation formula for MAE is as follows:

(22) $$MAE=\frac{1}{n}\sum_{i}^{n}\left|{\hat{y}}_{i}-y_{i}\right|$$

The calculation formula for RMSE is as follows:

(23) $$RMSE=\sqrt{\frac{1}{n}{\sum_{i}^{n}\left({\hat{y}}_{i}-y_{i}\right)}^{2}}\;$$

The calculation of RE, MAE, and RMSE is shown in the formula, where $\hat{y}$ represents the predicted result and y represents the actual value.

## 4. Experiments

### 4.1. Experimental Dataset

This article uses the publicly available laboratory data CS2_35 dataset of lithium-ion batteries, a key component of equipment, to conduct offline verification of the effectiveness and robustness of the RUL prediction model based on integrated learning. After offline verification is passed, the model is deployed and implemented on the maintenance support digital twin platform, with prediction parameters configured and real-time twin data of equipment connected to carry out online RUL prediction of equipment. The experimental dataset of lithium-ion battery CS2_35 is sourced from the Computer-Aided Life Cycle Engineering Laboratory (CALCE) at the University of Maryland. With the continuous advancement of technology, lithium-ion batteries have been widely used in fields such as weapons and equipment, aerospace, new energy vehicles, smartphones, etc. They are key components of equipment such as airplanes and missiles. As time and charge–discharge cycles increase, the performance of lithium-ion batteries will decrease to some extent [28]. As an energy storage and power supply unit, the performance degradation of lithium-ion batteries may cause equipment failures and safety accidents.

The experimental data of CS2_35 were measured at room temperature in the CALCE laboratory. Its rated capacity is 1.1 Ah. The specific experimental steps for obtaining the dataset are as follows: (1) In the experimental monitoring of lithium-ion battery charging, perform 1.5 A constant current charging on the lithium battery. During this process, monitor performance changes until the voltage rises to 4.2 V. (2) Keep the voltage at 4.2 V and continue charging until the current drops to 0.05 A. (3) During the experimental monitoring of discharge, a constant current of 1.35 A was used to discharge until the voltage dropped to 2.7 V. (4) The discharge rate during the experiment was maintained at 1 C [4].

In the charging and discharging experiment of CS2_35 lithium-ion battery, repeat the above steps until its capacity decreases to about 80% of the nominal capacity (i.e., the fault threshold). The nominal capacity is considered the EOL of the battery. The battery charge and discharge cycle data are stored in chronological order, with a negative discharge current value and a positive charge current value. The battery’s charge and discharge cycle can be determined based on the positive or negative current value, and the experimental data that needs to be obtained can be clarified [28].

The remaining capacity decreases with the charging and discharging cycles of the battery. The remaining capacity can be used as a degradation indicator for lithium-ion batteries. Therefore, the remaining capacity of the battery can be predicted to represent its RUL. The EOL cycle of battery CS2/35 is approximately 500. Therefore, 0–600 cycles of data are used as experimental and simulation data, 0–300 cycles are used for training and validation, and 300–600 cycles are used for result testing.

### 4.2. Time Series Conversion

This article converts the remaining capacity as a degradation index into a time series analysis problem. According to some experimental results and verification from reference [28], the time window step size is set to 10. This means that the next discharge cycle of the CS2_35 battery uses its 10 discharge cycle capacity data to predict the remaining capacity and convert it into time series data, that is, the 10 data of *t*, *t* + 1, *t* + 2, *t* + 3, *t* + 4, *t* + 5, *t* + 6, *t* + 7, *t* + 8, and *t* + 9 are used to predict time *t* + 10. In this article, the size of the time window is not studied or discussed, but is set to a fixed value of 10. The specific implementation steps are as follows:

(1) According to the usage scenario, if the charging and discharging processes are frequent, a small time window is given. Otherwise, a large time window is given. Due to the different usage scenarios of different types of lithium batteries, the charging and discharging data measured by testers are also different. It is necessary to give a time window based on the specific charging and discharging data of a particular battery and the user’s expected time window size.
(2) According to the time window, convert the raw data into time series data. Based on the size of the time window, obtain time series data with a time window size of L, which is ${RUL}_{t-1},\;{RUL}_{t-2},\;\ldots \;{RUL}_{t-L}$, where ${RUL}_{t}$ represents the time series data at time t and L represents the size of the time window.
(3) The original data conversion is completed, forming a time series dataset.

### 4.3. Training Parameter Settings

Firstly, the Excel file containing the data is read in through pd. read-excel and converted to a data frame type useful for pd. Then, the remaining capacity of the lithium battery is calculated by filtering based on column names, resulting in 881 battery capacity data points that vary over time. Take approximately 50% of the data before the failure of the lithium battery as the training and validation set, and approximately 50% as the testing set. The ratio of training and validation sets is approximately 9:1. Observing the degradation model of lithium batteries, it can be seen that they work for about 600 cycles before failure. Therefore, the 0–270 cycle data is used as the training set, the 270–300 cycle data is used as the validation set, and the 300–600 cycle data is used as the test set.

The basic learner parameter settings are as follows:

(1) Related Vector Machine: The number of iterations (n_estimators) is 100, and the error precision (learning_error) is 0.0001.
(2) Random Forest: It has 160 iterations (n_estimators) and an error of 0.001. Error 0.001 is a very small threshold, indicating that the model is expected to reach a very stable state before stopping.
(3) Elastic Network: The number of iterations: 1000; the proportion of L1 norm penalty term: 0.001; and the parameter penalty in L2: 0.001. This setting is highly biased towards L2 regularization (ridge regression), implying that prior knowledge or experimental findings suggest that features in the data are likely to be highly correlated, and we want to preserve all features but shrink their coefficients, rather than performing feature selection (the main role of L1). “L1_ratio = 0.001” is almost a ridge regression model designed to address collinearity issues and improve model stability. “L2_alpha = 0.001” is a very small regularization strength. Combined with L1_ratio = 0.001, this constitutes a very mild regularization strategy. The model mainly relies on the L2 penalty to handle small collinearity, while hardly applying sparsity 0.001 can provide the best generalization performance.
(4) Autoregressive model: The order of the difference equation is 21.
(5) Long Short-Term Memory network: 2 layers, 128 hidden layer neurons.

Genetic Algorithm Integrated Basic Learner:

The weights a, b, c, d, and e of each basic learner are the real-valued encoding of any number between 0 and 1.

Initialize the population size to 800.

Set the fitness function to:

(24) $${\frac{1}{n}\sum_{i=1}^{n}\;(y_{i}-ay_{i}^{1}-by_{i}^{2}-cy_{i}^{3}-dy_{i}^{4}-ey_{i}^{5})}^{2}$$

And minimize the fitness function where n represents the data size for training the ensemble algorithm. The $y_{i}^{j}(j=1,2,3,4,5)$ represents the prediction result of sequence i on the base learner j. $y_{i}$ represents the true value of sequence i, and a, b, c, d, and e represent the weights of each base learner, respectively.

Continuously selecting, crossing, and mutating, the evolutionary probability is 0.01. If the update round reaches the set maximum value of 800, it will terminate.

Using GA, multiple rounds of experiments and verification were conducted with an initial population of 100, a maximum iteration of 800, and a mutation probability of 0.01. After 800 iterations, the fitness value can be minimized, and the result is 4.97513239 × 10^−5^.

### 4.4. Experimental Results

We simulated and trained the time series dataset of CS2-35 battery charging and discharging cycles, and obtained 5 prediction results. Then, GA was used to find and select the optimal weights for each learner. The final prediction model was obtained. Among them, the optimal weights for each basic learner are shown in Table 1.

**Figure 5:**
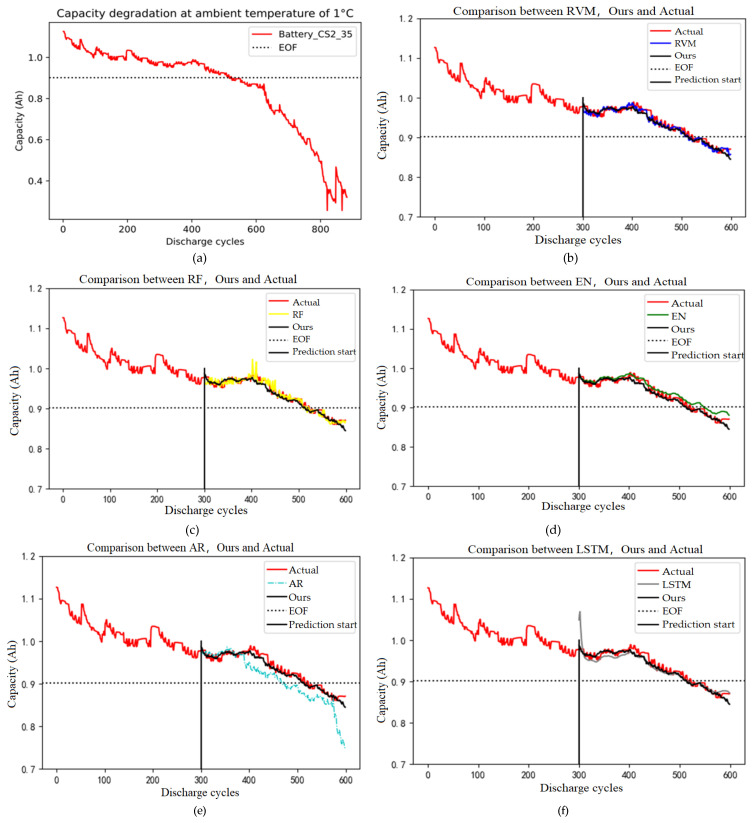
Experimental result. (**a**) Capacity degradation of Battery_CS2_35; (**b**) comparison between RVM, ours, and actual; (**c**) comparison between RF, ours, and actual; (**d**) comparison between EN, ours, and actual; (**e**) comparison between AR, ours, and actual; and (**f**) comparison between LSTM, ours, and actual.

From Table 1, it can be seen that the weights of the five basic learners are all greater than 0, indicating that each basic learner contributes to the final integrated learning method. It can be seen that by integrating multiple basic prediction methods, the effect may be better than that of a single basic learner. The capacity degradation of the CS2_35 battery is shown in Figure 5a. Figure 5b–f show the comparison results between the integrated learning method and a single base learner for the CS2_35 battery after 300–600 cycles.

In Figure 5b, it can be seen that the integrated model can fit the actual degradation curve of the battery well, and its fitting degree is higher than that of the RVM method. In Figure 5c, it can be seen that the Random Forest prediction results can basically fit the actual degradation curve of the battery. There is a significant difference between the 400–450 charge and discharge cycle periods and the actual degradation curve, while the ensemble learning method can fit the actual degradation curve of the battery well in 300–600 charge and discharge cycle periods, with higher accuracy. In Figure 5d, it can be seen that the predicted results of the EN are slightly higher than the actual degradation values, while the ensemble learning method can fit the actual degradation curve of the battery well in 300–600 charge–discharge cycles, and the accuracy of the ensemble model prediction is better than that of the Elastic Net.

In Figure 5e, it can be seen that the prediction results of the AR model can fit the degradation curve of the battery well in the 300–380 charge and discharge cycle periods. In the 380–600 charge and discharge cycle periods, the prediction results are lower than the actual degradation value, with a capacity deviation of about 0.03. However, the ensemble learning method can fit the actual degradation curve of the battery well in the 300–600 charge and discharge cycle periods, and the prediction accuracy of the ensemble learning method is better than that of the AR model. The poor predictive performance of AR is due to its purely linear nature and strict assumption of stationary data (i.e., mean and variance remain constant over time). In contrast, real-world time series data (e.g., stock prices, equipment vibration signals, and power loads) often exhibit complex nonlinear patterns (such as periodic abrupt changes, state transitions) and non-stationarity (e.g., trends, drifts). The AR model can only rely on pre-defined, fixed-window (order p) historical points. Unlike LSTM, it lacks the ability to selectively retain or forget long-term historical information through internal cell states. Standard AR models use only the historical values of the target sequence as input, unable to leverage rich external information, resulting in limited predictive capability in complex systems. Parameter estimation methods like least squares in AR models are highly sensitive to outliers, where a single anomaly can significantly distort all coefficient estimates. The “poor” performance of AR is relative to more sophisticated and flexible modern models when handling complex real-world data. AR models remain highly efficient, interpretable, and computationally inexpensive for scenarios where data is approximately linear, stationary, and low-noise. They also serve as the foundation for building more complex models (e.g., ARIMA, VAR). If the relationships are simple and trends are clear, AR/ARIMA may be the optimal choice (fast, easy to interpret).

In Figure 5f, it can be seen that the prediction results of the LSTM model can fit the degradation curve of the battery well in 300–600 charge and discharge cycles, but the prediction results are all low, with a deviation of about 0.01. However, the ensemble learning method can fit the actual degradation curve of the battery well in 300–600 charge and discharge cycles, and the prediction accuracy of the ensemble learning method is better than that of the LSTM model. Therefore, when comparing the degradation prediction results with the actual degradation curve of the battery, ensemble learning methods are significantly superior to RVM, RF, EN, AR, and LSTM methods.

Further analysis was conducted using RE, MAE, and RMSE to compare the differences in prediction results between ensemble learning methods and base learners, as shown in Table 2.

Table 2 shows the RE, MAE, and RMSE values for each basic learner and ensemble learning used to predict the remaining capacity of the CS2_35 battery. The experimental results show that compared with single methods based on RVM Bayesian, RF ensemble tree, EN (Elastic Net), AR random model, and LSTM-ANN, ensemble learning methods have smaller RMSE. The RMSE of RVM is 0.0074, RF is 0.0110, EN is 0.0151, AR is 0.0322, LSTM is 0.0154, and the ensemble learning method is 0.00483. The RMSE of the ensemble learning method decreases by a maximum of 0.0274 and a minimum of 0.00261, and the RE of the ensemble learning method is 0.0207, which is lower than other models. The MAE is 0.0061, indicating that the ensemble learning method has stronger robustness and higher prediction accuracy. The degradation curve of the remaining capacity of the CS2_35 battery in ensemble learning reaches the end of its lifespan earlier than in reality. Therefore, by predicting the RUL of the battery in advance, issuing warnings, and taking preventive maintenance measures such as early replacement or on-site maintenance before the actual service life of the battery ends, it can effectively prevent the occurrence of life, property, and other dangerous events caused by equipment system failures caused by lithium-ion battery failures.

To further validate the effectiveness of ensemble learning methods, experiments were conducted on the CS2F35 dataset to compare ensemble learning with DAE-LSTM [29], transfer learning-based DAE-LSTM [29], and the DEGWO-MSVR model [30] proposed in recent years. The results are shown in Table 3.

In the experimental results in Table 3, it can be seen that the RMSE of the ensemble learning method is 0.0207, which is the highest decrease of 0.0226 and the lowest decrease of 0.0075 compared to other methods. The RE of the ensemble learning methods is 0.0207, which is lower than that of the DAE-LSTM, transfer learning-based DAE-LSTM, and DEGWO-MSVR models. The MAE of the ensemble learning method is 0.0061, which is lower than that of the DAE-LSTM, transfer learning-based DAE-LSTM, and DEGWO-MSVR models. Therefore, the ensemble learning method has stronger robustness and higher prediction accuracy.

### 4.5. Engineering Implementation

**Figure 6:**
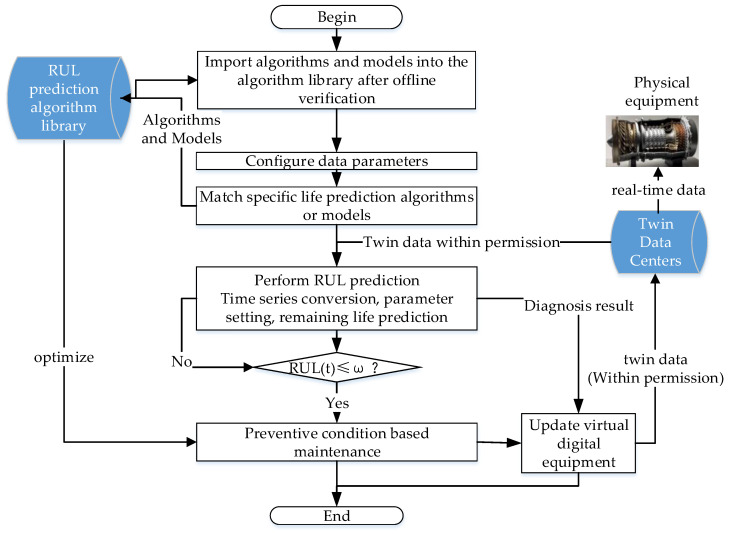
Implementation process of the twin data-driven RUL prediction project.

The engineering implementation process of the online real-time application of the RUL driven by twin data is shown in Figure 6. Firstly, the RUL prediction algorithm for each working condition or combination of working conditions verified by historical data will be imported into the maintenance support digital twin platform to form a RUL algorithm library. Secondly, based on the characteristics of the equipment and the twin data, configure the RUL calculation parameters corresponding to the equipment, subsystem, or component. Then, based on the characteristics of the equipment, subsystems, or components, match the corresponding RUL prediction algorithm or model from the RUL algorithm library. Next, based on the access permissions, access the authorized equipment twin data stored in the twin data center (sensitive equipment data is authorized through the maintenance support digital twin platform to form data access permissions), load it into the RUL prediction algorithm and model, and execute the RUL prediction process, including time series conversion, training parameter setting, and remaining life calculation steps. If RUL (t) > ω (RUL threshold), it will operate normally and continue to monitor the RUL of the equipment based on real-time twin data. If RUL (t) ≤ ω, initiate preventive visual maintenance work and update virtual digital equipment simultaneously.

**Figure 7:**
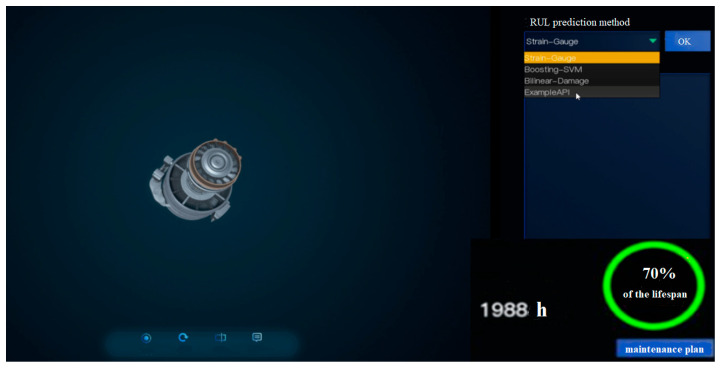
Online RUL prediction software diagram.

The user interface of the online real-time RUL prediction software based on digital twins is shown in Figure 7.

The RUL threshold is determined based on the equipment degradation curve, maintenance time, spare parts ordering, and supply time. The timing of maintenance based on the situation is generally set between the beginning of degradation and the occurrence of faults. The specific timing needs to consider the average maintenance time, spare parts ordering time, and spare parts supply time, in order to avoid faults as much as possible, maximize equipment operation time, minimize downtime, and minimize support costs. When the spare parts inventory is insufficient, in order to maximize equipment utilization and reduce support costs, the maintenance timing is determined by the RUL threshold ω, which is related to the replacement time of degraded parts, spare parts ordering time, spare parts supply time, and in situ repair time. When the spare parts inventory is available, there is no need to place an order for spare parts. The threshold value of the RUL of the equipment for initiating maintenance based on the situation, ω, is shown in Equation (25).

(25) ω=treplace+torder+tsupply, insufficient spare parts and trepair≥ torder+tsupply,and can not be repaired in situtrepair sufficient spare parts, can not be repaired in situ trepair ≥ tordering+tsupply treplace sufficient spare parts 

**Figure 8:**
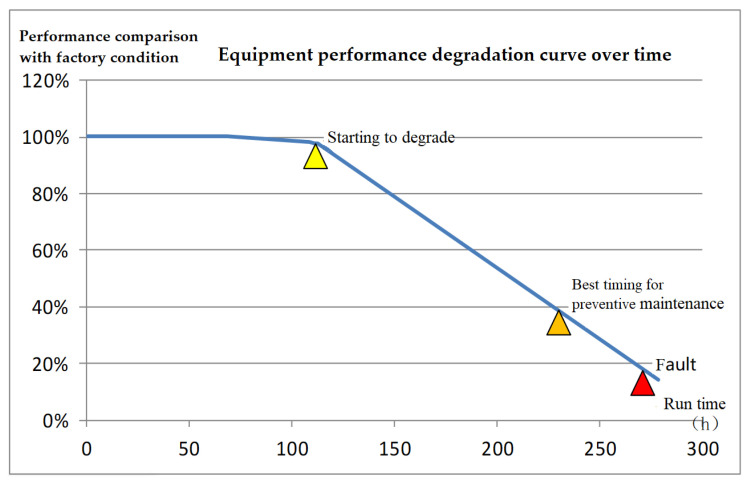
Performance degradation curve of certain equipment.

In the formula, t_repair_ refers to the repair time of degraded parts, which generally requires the shortest replacement and repair. When there are sufficient spare parts, it is generally used. The t_order_ refers to the spare parts ordering time, and the t_supply_ refers to the supply time after the spare parts are ordered. When the RUL (t) of the degraded component is less than or equal to ω, initiate the condition-based maintenance work. When spare parts are in stock, they can be used directly, and the equipment downtime is the replacement time of degraded parts, with the shortest downtime. When the spare parts inventory is out of stock, the threshold for RUL is ω, which is t_replace_ + t_order_ + t_supply_. At this point, it is necessary to order spare parts and then carry out repair work. When the spare parts inventory is out of stock, t_repair_ < t_order_ + t_supply_, degraded parts can be repaired in situ, and the RUL threshold is ω, which is t_repair_. At this time, in situ repair work should be carried out as appropriate. When the spare parts inventory is out of stock, t_repair_ < t_order_ + t_supply_, and the degraded parts cannot be repaired in situ, the RUL threshold is ω, which is t_replace_ + t_order_ + t_supply_. Depending on the situation, repair work should be carried out. Figure 8 shows the degradation curve of certain equipment and the timing of condition-based maintenance. When the remaining lifespan threshold is ω, conducting condition-based maintenance work can maximize the utilization of equipment performance while reducing equipment downtime and maintenance support costs.

The digital twin platform provides the function of selecting RUL prediction methods, and the software can select specific prediction algorithms for equipment and key components, either individually or in combination. Users choose RUL prediction algorithms built into the algorithm library based on the characteristics of the equipment structure and components. Next, configure the prediction parameters. The digital twin platform software displays a parameter list of all the twin data of the equipment. Through the selection box, the engineering implementation personnel configure the predicted parameters based on the specific parameter data usage requirements of the selected RUL prediction algorithm and model. The digital twin platform software loads the relevant twin data of the configured parameters into the RUL prediction algorithm and model for RUL prediction. When the RUL(*t*) of equipment, key components, or subsystems is ≤ω, carry out maintenance as needed, determine maintenance strategies, develop maintenance plans, and carry out early maintenance work.

When feasible, we prioritize Remaining Useful Life prediction models with good interpretability (such as linear regression, decision trees, and rule-based models), or by combining ensemble methods (such as Random Forests, XGBoost) to provide feature importance analysis. For complex models such as deep learning, we use methods such as LIME (Local Interpretable Model agnostic Explanations) or SHAP (Shapley Additive exPlans) to provide local feature contribution explanations for individual prediction samples, helping to understand the basis for specific prediction results. For time series data, the digital twin platform software dynamically displays the changes in feature importance over time, highlighting the changes in key indicators at different stages of equipment degradation. It uses charts (such as bar charts, heat maps, and trend lines) to visually present feature importance.

The digital twin platform software combines model prediction results with feature analysis to generate concise natural language descriptions (such as: “The current vibration amplitude has increased by 30% compared to last week, approaching the historical fault threshold. It is recommended to check the bearing wear condition”). Based on the predicted results and combined with the domain knowledge base or expert rules, the software provides specific maintenance recommendations (such as: “It is recommended to replace component A within 7 days and check the associated component B”).

The digital twin platform software constructs an interactive decision support interface to display RUL prediction results based on “numerical + confidence interval”, real-time monitoring, historical comparison of key features, and visualization of degradation stages (such as health, decline, and severe faults). The software allows users to adjust input parameters (such as load changes) and simulate their impact on lifespan.

Building a real-time online RUL (RUL) prediction system, especially when implemented on a large scale, will face a series of systemic challenges from computing power, infrastructure, and real-world deployment. The main challenges of computing power and algorithms are real-time performance (completing model inference within milliseconds), model update overhead within seconds, and proportional CPU/GPU/memory requirements. The hardware infrastructure challenges include three aspects: network and latency (edge cloud data transmission bandwidth and latency bottlenecks, model splitting overhead), heterogeneous resource scheduling (efficient allocation of heterogeneous resources such as CPU, GPU, memory, etc., achieving “peak shaving and valley filling” and training reuse), and edge device limitations (limited device computing, memory, and storage, requiring deployment of lightweight models). The implementation of deployment faces challenges such as deviation of online data distribution from the training set due to changes in equipment operating conditions and environment, decreased model performance, difficulty in cross-departmental or enterprise data sharing, complex integration with existing SCADA, MES, and other systems, and high cost of building high-fidelity digital twins. Its feasibility highly depends on specific scenarios, technology selection, and cost investment. Generally, a cloud edge collaborative architecture is adopted, where models (such as BTCAN and GT MR Net) are compressed to the extreme and deployed directly on edge devices or gateways. Federated learning is used, where each device trains locally and only uploads model parameters (such as gradients) to the cloud for aggregation, generating a global model.

To ensure the implementation effect of online remaining service life prediction for basic digital twins, it is necessary to first clarify the scenario and requirements, pursue ultimate real-time performance for key components (edge light weighting), and globally and continuously optimize the strongest model of the equipment system (cloud edge collaboration/federated learning). Secondly, by combining physical mechanisms with data-driven approaches, we aim to enhance robustness under small sample and variable operating conditions. Simultaneously, design continuous/online learning mechanisms (such as online PINN) enable the model to adapt to changes in device degradation patterns, pay attention to data engineering and uncertainty quantification, and provide confidence intervals for prediction results to provide risk references for decision-makers. Finally, the same high-value and battery well data-based production line or equipment type will be piloted to validate the technical route and quantify the economic benefits, and then gradually promoted in stages.

## 5. Conclusions

This paper introduces digital twin technology in the prediction of RUL, proposes a digital twin-based model for predicting the RUL of equipment, and analyzes the data-driven process of predicting the RUL of twins. Aiming at the low accuracy and robustness of current single data-driven methods for predicting RUL, an ensemble learning method for predicting RUL is proposed. The ensemble learning method integrates commonly used time series RUL methods (such as RVM, RF, EN, AR, and LSTM) into basic trainers and uses genetic algorithms to calculate the optimal weights of each basic trainer, forming an ensemble learner to predict the RUL of equipment and its components. The accuracy and robustness advantages of the method were validated using the CS2_35 battery dataset from the CALCE laboratory. Finally, an online engineering implementation process for predicting RUL based on digital twins was presented, along with a method for calculating the RUL threshold. The experiment shows that the RE of the ensemble learning method is 0.0207, MAE is 0.0061, and RMSE is 0.0048, all of which are lower than SVM, RF, EN, AR, LSTM, DAE-LSTM, DEGWO-MSVR, and other models, and have advantages in accuracy and robustness. The research results of this article can be used as input for maintenance decision-making. Based on accurate residual service life thresholds, the optimal maintenance timing can be determined, and the optimal maintenance strategy can be adopted to implement precise equipment support.

This article applies standard datasets for RUL prediction validation. However, in practical industrial applications, the accuracy of RUL prediction is highly correlated with environmental factors such as temperature, humidity, and load conditions. Therefore, in further work, we need to optimize the RUL prediction model based on the physical degradation mechanism of industrial equipment and the dependence of environmental parameters, integrate environmental factors into the prediction model, and further establish an environment-dependent RUL prediction model. Then, environmental data and real-time monitoring data are used as inputs to calculate and predict in the RUL prediction model, ensuring the universality and accuracy of the model’s application in industrial equipment. Future work can explore cross-domain validation, incorporating uncertainty quantification into predictions, and adapting models to dynamic operating conditions. Additional indicators, such as the coefficient of determination (R^2^), will be added to the model evaluation to further demonstrate the model’s goodness of fit and predictive ability. The RUL prediction method combining physical mechanism models with data-driven machine learning is also something we need to further study. The RUL prediction method combining physical mechanism models with data-driven machine learning can reduce the performance overhead of online digital twin prediction, improve online real-time performance, ensure minimal equipment downtime, and reduce maintenance costs.

## Figures and Tables

**Table 1:** Basic learners’ weights.

Basic Learners	Weights
RVM	0.312403
RF	0.04751
EN	0.310046
AR	0.202221
LSTM	0.128759

**Table 2:** RE, MAE, and RMSE comparison.

Methods	RE	MAE	RMSE
RVM	**0.0257**	**0.0034**	0.0074
RF	0.0571	0.0080	0.0110
EN	0.0412	0.0127	0.0151
AR	0.1285	0.0240	0.0322
LSTM	0.1122	0.0102	0.0154
Ours	**0.0207**	0.0061	**0.0048**

**Table 3:** RE, MAE, and RMSE comparison.

Model	RE	MAE	RMSE
DAE-LSTM [29]	0.1531	0.0237	0.0274
Transfer Learning-based DAE-LSTM [29]	0.0823	0.0096	0.0123
DEGWO-MSVR [30]	0.0224	0.0197	0.0237
Ours	**0.0207**	**0.0061**	**0.0048**

## Data Availability

The data can be accessed from this manuscript.

## Abbreviations

The following abbreviations are used in this manuscript:

RU	Remaining Useful Life
PHM	Fault diagnosis, Prediction, and Health Management
DL	Deep learning
RVM	Relevance Vector Machine
RF	Random Forest
AR	Autoregressive
EN	Elastic Net
LSTM	Long Short-Term Memory
GA	Genetic Algorithm
RE	Relative Error
MAE	Mean Absolute Error
RMSE	Root Mean Square Error
ANN	Artificial Neural Network
CNN	Convolutional Neural Network
RNN	Recurrent Neural Network
DAE-LSTM	Denoising AutoEncoder–Long Short-Term Memory
DEGWO-MSVR	Differential Evolution Grey Wolf Optimizer–Multioutput Support Vector Regression
CALCE	Computer-Aided Life Cycle Engineering Laboratory
PLC	Programmable Logic Controller
CNC	Computer Numerical Control
CAN	Controller Area Network
OPC UA	OLE for Process Control Unified Architecture
RTU/TCP	Remote Terminal Unit/ Transmission Control Protocol
LIME	Local Interpretable Model agnostic Explanations
SHAP	Shapley Additive exPlans
LASSO	Least Absolute Shrinkage and Selection Operator
